# Supplementary material for: Characterization and Modeling of Free Volume and Ionic Conduction in Multiblock Copolymer Proton Exchange Membranes
Source: Polymers (Basel). 2022 Apr 21;14(9):1688. doi: 10.3390/polym14091688 (PMC9100545; doi:10.3390/polym14091688)
Supplement: Supplementary file 1 [file polymers-14-01688-s001.zip › polymers-1657010-supplementary.pdf]

Supplementary material to

Characterization and Modelling of Free Volume and Ionic Conduction in Multiblock Copolymer Proton Exchange Membranes

Mahmoud M. Gomaa[1], Arturo Sánchez-Ramos[2], Nieves Ureña[3], María Teresa Pérez-Prior[3], Belén Levenfeld[3], Pablo A. García-Salaberri[2,*], Mohamed R.M. Elsharkawy[1]

1 Department of Physics, Minia University, P.O. Box 61519 Minia, Egypt

2 Department of Thermal and Fluids Engineering, Universidad Carlos III de Madrid, Leganés 28911, Spain

3 Department of Materials Science and Engineering and Chemical Engineering, Universidad Carlos III de Madrid, Leganés 28911, Spain

* Corresponding author: **Pablo A. García-Salaberri**

Fluid Mechanics Research Group, Dept. of Thermal and Fluids Engineering

Universidad Carlos III de Madrid

Avda. de la Universidad, 30, 28911 Leganés, Madrid, Spain

Email address: pagsalab@ing.uc3m.es

URL: http://fluidosuc3m.es/people/pagsalab

URL: http://www.salaberri.com

Pho: +34 91 624 9407

1. PALS setup

The *o*-Ps lifetime was determined at various times at 80% RH in order to determine the minimum time necessary to achieve a complete saturation of the vapor-equilibrated membranes. A full PALS spectrum was collected every 2 hours for a total run of 36 hours. As shown in Figure 1, the *o*-Ps lifetime increased gradually with the exposure time until 20 h and then remained constant. The same procedure was used for other membranes and operating conditions.

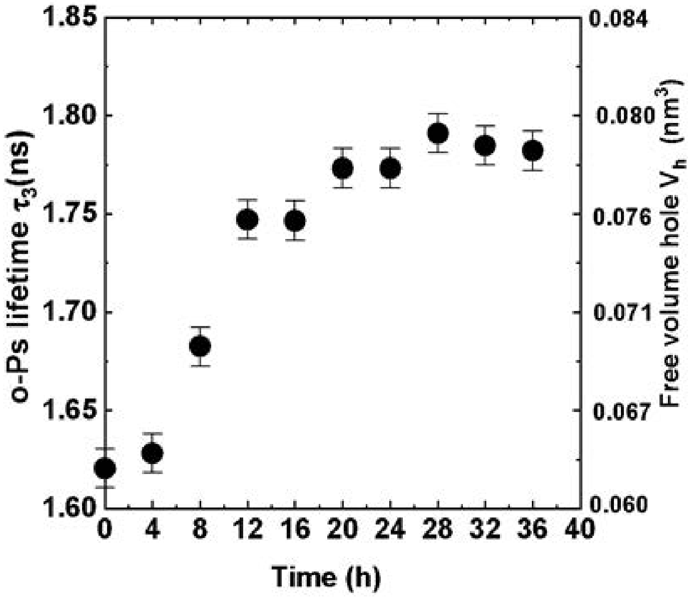

S1: Evolution of the *o*-Ps lifetime and free volume, V_h, with time during the setup of the PALS measurements of the copolymer PEMs at 80% RH.

2. Thermal expansion coefficient

The thermal expansion coefficient of PEMFC components must be closely controlled to reduce strains produced by differential thermal expansion. The thermal expansion coefficient associated to the free volume of the membranes can be calculated as

$$\beta = \frac{1}{V_{h,o}} \frac{\Delta V_h}{\Delta T} \tag{1}$$

where $V_{h,o}$ is the *o*-Ps hole size corresponding to the intercept with the *y*-axis. As shown in Figure 2, the thermal expansion coefficients of the copolymer PEMs are $\beta_1 = 1.37 \times 10^{-3}$ °C^{-1} (SPES 1), $\beta_2 = 2.18 \times 10^{-3}$ °C^{-1} (SPES 2) and $\beta_3 = 2.95 \times 10^{-3}$ °C^{-1} (SPES 3).

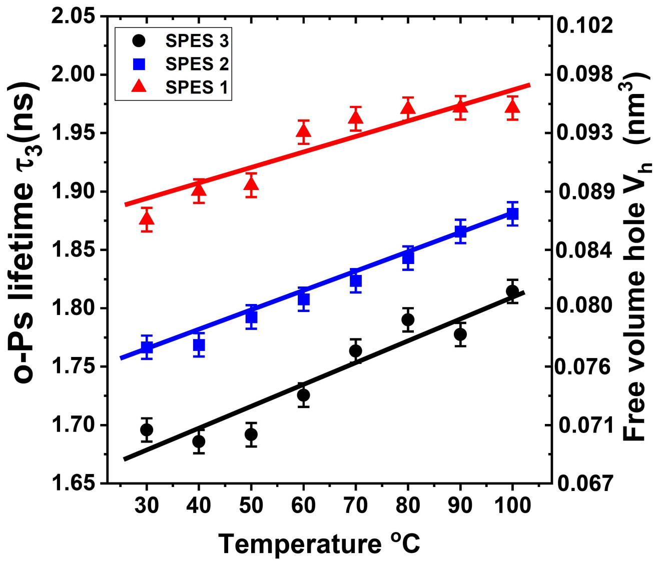

S2: Variation of the *o*-Ps lifetime and free volume, V_h, with temperature, T. The thermal expansion coefficient, β, can be determined from the almost linear relationship V_h-T.

3. Bilinear interpolation

The contours of the characteristic pore radius, r_c, and the standard deviation, σ, of the fitted log-normal distributions of the copolymer membranes as a function of relative humidity, RH, and temperature, T, are shown in Figure 3. The distributions were determined using bilinear interpolation from the measured PALS data. r_c and σ increases with RH and T, especially in the case of SPES 3.

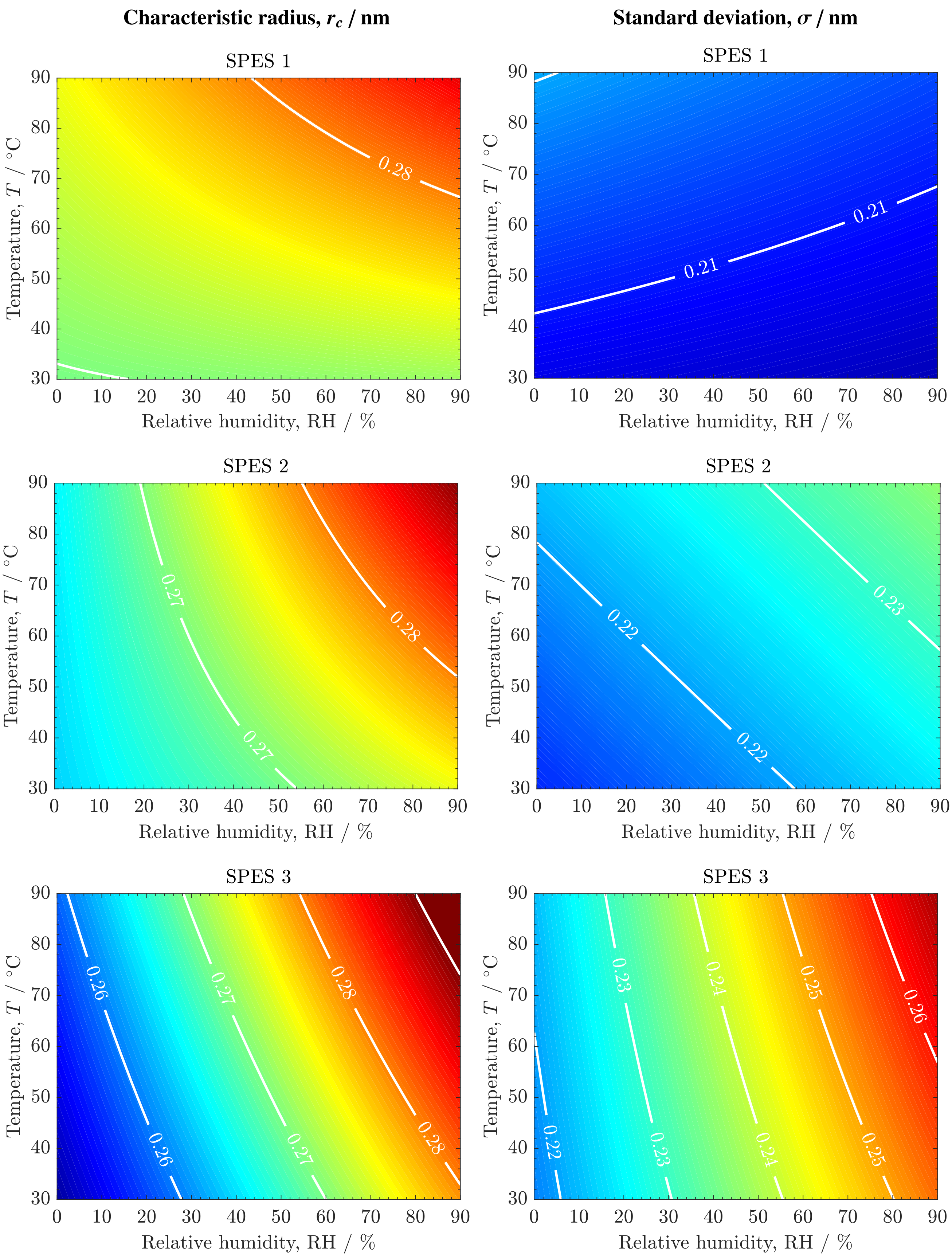

S3: Variation of the characteristic pore radius, r_c, and the standard deviation, σ, as a function of relative humidity, RH, and temperature, T, of the copolymer membranes.

4. Proton concentration and surface charge density

The volume average and the fluid average proton concentration, $C^f_{\mathrm{H^+}}$ and $C^{\mathrm{avg}}_{\mathrm{H^+}} = \phi_v C^f_{\mathrm{H^+}}$, and the surface charge density, σ, as a function of RH predicted by the bundle-of-tubes model are shown in Figure 4. $C^f_{\mathrm{H^+}}$ in the hydrated tubes remains almost constant with RH due to the electroneutrality condition. However, $C^{\mathrm{avg}}_{\mathrm{H^+}}$ increases with RH due to the overall increase of the water volume fraction (i.e., higher number of hydrated ionic tubes per unit volume). Consequently, σ increases with RH (i.e., it becomes more negative) due to the decrease of the hydrated specific surface area per unit of fluid volume, a_f, caused by the growth of the average pore radius. Note that $C^f_{\mathrm{H^+}} \sim -\sigma a_f / F \sim$ const.

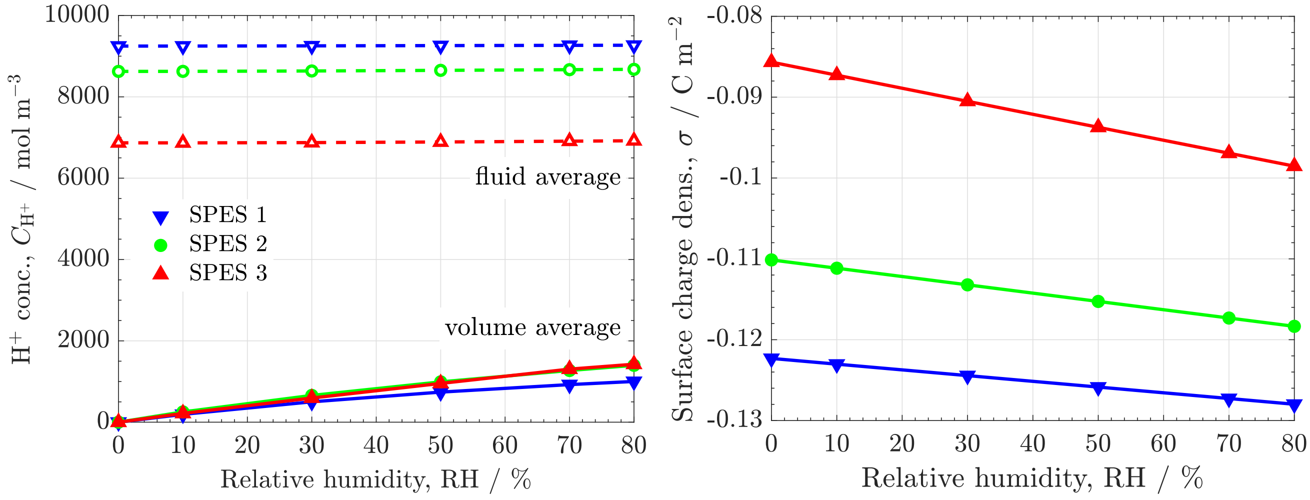

S 4: Variation of the volume average and the fluid average proton concentration, $C_{\mathrm{H^+}}$, and the surface charge density, σ, as a function of relative humidity, RH, predicted with the bundle-of-tubes model for the three copolymer membranes.
